# An Incidental Pancreatic Finding at 18F-Choline PET/CT: Chronic Mass-Forming Pancreatitis

**DOI:** 10.3390/diagnostics11081490

**Published:** 2021-08-17

**Authors:** Laura Evangelista, Alessandro De Pellegrin, Rossano Girometti, Gianluca Cassarino, Francesco Giacomuzzi, Marco Rensi

**Affiliations:** 1 Nuclear Medicine Unit, Department of Medicine DIMED, University of Padova, 35128 Padova, Italy; giancassa@hotmail.it; 2 Department of Pathology, University Hospital S. Maria della Misericordia, 33100 Udine, Italy; alessandro.depellegrin@asufc.sanita.fvg.it; 3 Institute of Radiology, Santa Maria della Misericordia Academic Medical Centre, 33100 Udine, Italy; rossano.girometti@asufc.sanita.fvg.it; 4 Department of Medicine, University of Udine, Santa Maria della Misericordia Academic Medical Centre, 33100 Udine, Italy; 5 Nuclear Medicine Unit, University Hospital S. Maria della Misericordia, 33100 Udine, Italy; Francesco.giacomuzzi@asufc.sanita.fvg.it (F.G.); marco.rensi@asufc.sanita.fvg.it (M.R.)

**Keywords:** fluorocholine, PET/CT, pancreatitis, magnetic resonance imaging, histopathology

## Abstract

We present a case of a chronic mass-forming pancreatitis (CMFP) detected by 18F-choline (FCH) PET/CT in a male affected by prostate cancer. FCH PET/CT scan showed a focal uptake in the uncinate process of the pancreas, later diagnosed as a CMFP at biopsy. Although the physiological distribution of FCH in the pancreas, a careful interpretation of the images in this area is warranted.

**Figure 1:**
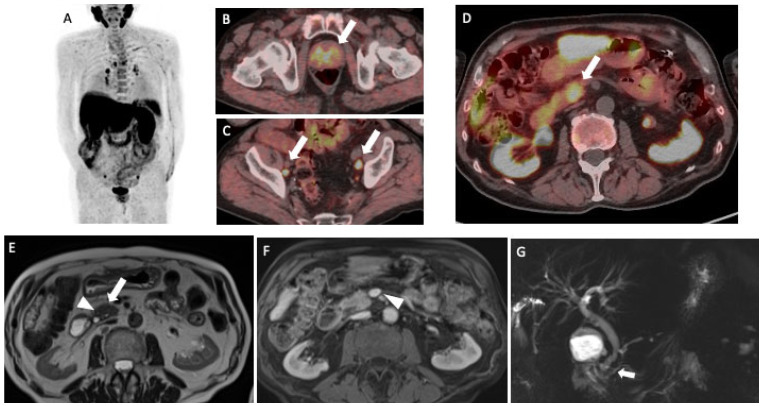
A male patient with prostate cancer underwent 18F-Choline (FCH) PET/CT for the suspicion of lymph node recurrence during hormonal therapy. He was not submitted to local therapies (i.e., surgery or radiotherapy) for the treatment of the primary prostate lesion. The patient has, in addition, a history of diabetes mellitus type II and hypertension. PET/CT detected multiple areas of focal FCH uptakes ((**A**); maximum intensity projection—MIP) in prostate gland (arrow, (**B**)) and abdominal-pelvic lymphadenopathies (arrows, (**C**)), compatible with recurrent prostate cancer. Moreover, a focal tracer uptake was shown in the uncinate process of the pancreas with a maximum standardized uptake value (SUVmax) equal to 13.6 (arrow, (**D**)). As already stated by Schillaci et al. [1], FCH may physiologically show a moderate-to-high uptake in the liver and the pancreas. However, the presence of a focal uptake should be further investigated in order to make a differential diagnosis with a malignant or benign lesion. A subsequent MRI showed no definite lesion but rather a slight “mass-like” enlargement of the pancreatic head, as visible on the axial single-shot turbo-spin echo T2-weighted image (arrow, (**E**)). Some micro-cystic areas were appreciated in the pancreaticoduodenal groove (arrowhead, (**E**)). The absence of lesions was confirmed on the post-contrast pancreatic phase (axial gradient echo volumetric fat saturated image), in which pancreatic parenchyma appears homogeneous, with no solid tissue extending towards major vascular structures (arrowhead, (**F**)). Of note, the cholangiopancreatography sequence showed a normal-sized but irregular main pancreatic duct, with no cephalic structures (arrow, (**G**)). Overall, subtle morphological changes and ductal abnormalities supported the diagnosis of CMFP. The Carbohydrate antigen 19–9 (CA19.9) was normal (17.0 UI/mL). However, after one month from MRI and FCH PET/CT, the patient was submitted to an endoscopic ultrasound (EUS) exam and biopsy, based on the suggestion of the multidisciplinary team.

**Figure 2:**
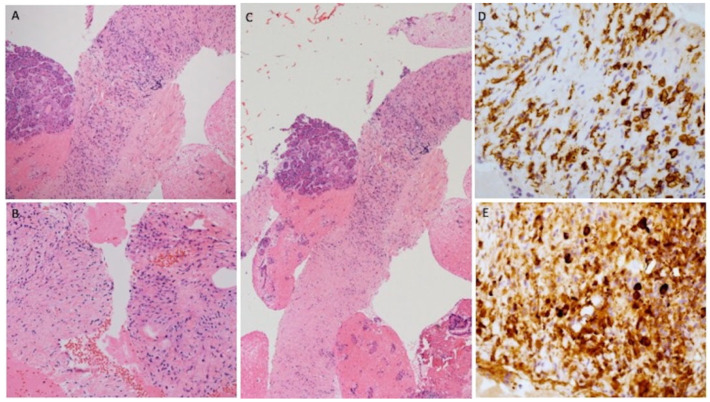
The histological examination demonstrated fragments of sclerotic stroma in storiform fashion, in which inflammation cells (most lymphocyte and plasma cells) were recognizable ((**A**–**C**); Hematoxylin and Eosin stains at different magnifications). Moreover, the immunostaining for CD38 highlighted the plasma cells population (**D**) and immunostaining for IgG4 showed a considerable amount of specific immunoglobulin in diagnostic range for type 1 autoimmune pancreatitis (**E**). The CMFP represents the 10–30% of chronic pancreatitis and the differential diagnosis with pancreatic cancer is very difficult [2]. MRI has a high diagnostic accuracy in the diagnosis of chronic pancreatitis with a sensitivity and specificity, respectively, of 78% and 96% [3]. Gu et al. suggest using 18F-FDG PET/CT combined with CA19–9 to differentially diagnose pancreatic cancer from CMFP [4]. EUS is a very sensitive examination to detect pancreatic masses and can provide useful information in cases where conventional radiologic workup remains inconclusive [5]. Thanks to its high-resolution images, EUS is more precise than MRI and CT for the diagnosis of small pancreatic lesions. However, its specificity remains low (about 50%) since most lesions showed unclear echoic signs. Probably, the association of EUS with contrast enhancement and elastography can increase the overall accuracy [6,7]. Choline plays an important role in the structure and the function of biological membranes and is essential for the synthesis of phospholipids [8]. The distribution of FCH is physiological in the pancreas as in the liver, spleen, salivary, and lacrimal glands [1]. The uptake of choline is also present in many cancers and in the immune cells as macrophages, present in inflammatory processes [9,10,11]. This is the first case of CMFP detected by FCH. We cannot recommend the use of FCH PET as an alternative diagnostic imaging in autoimmune pancreatic processes, but we strongly suggest careful interpreting PET-imaging (especially in case of focal tracer uptake in the pancreas). Furthermore, images from hybrid PET/MRI scanners [8] should be assessed, since they can provide complete information by using a single examination.
